# The development and initial evaluation of the Diarrhoea Management Diary (DMD) in patients with metastatic breast cancer

**DOI:** 10.1007/s10549-020-05798-w

**Published:** 2020-07-27

**Authors:** Helena Harder, Valerie M. Shilling, Shirley F. May, David Cella, Peter Schmid, Lesley J. Fallowfield

**Affiliations:** 1 Sussex Health Outcomes Research and Education in Cancer (SHORE-C), Brighton and Sussex Medical School, University of Sussex, Brighton, UK; 2 Department of Medical Social Sciences, Feinberg School of Medicine, Northwestern University, Chicago, IL USA; 3 Centre for Experimental Cancer Medicine, Barts Cancer Institute, Queen Mary University London, London, UK

**Keywords:** Chemotherapy-induced diarrhoea, Adverse effects, Measurement, Patient-reported outcomes, Quality of life, Supportive care, Self-management

## Abstract

**Purpose:**

Chemotherapy-induced diarrhoea (CID) is a common, but often underreported problem in patients with breast cancer that has a profound effect on quality of life. It is best measured from a patient’s perspective, but tools are limited. The aim of this study was to develop and evaluate the Diarrhoea Management Diary (DMD), a self-report measure to assess CID, use of self-management strategies and treatment adherence.

**Methods:**

The DMD was constructed using an iterative process of instrument development: concept elicitation (literature review), item generation and reduction (cognitive debriefing), and pilot testing in the target population. After translation into eight languages, the DMD was used in an international randomised trial for women receiving lapatinib and capecitabine for metastatic breast cancer with or without prophylactic octreotide. Patterns of missing data and sensitivity to change were examined.

**Results:**

The understandability and completeness of the 8-item DMD was confirmed in cognitive interviews and pilot testing. Practicability of the DMD was evaluated in 62 women with metastatic breast cancer (median age 57). Up to 68% reported CID at any given time-point, and 19% had diarrhoea at each time-point. Patients also described efficacy of different strategies for diarrhoea management. Missing data were associated with study discontinuation. DMD missing item response was 0.9%. Sensitivity to change was good at most assessment points.

**Conclusions:**

Although further psychometric testing is recommended, initial evaluation of the DMD showed good content validity and practicability in international research with cancer patients.

**Electronic supplementary material:**

The online version of this article (10.1007/s10549-020-05798-w) contains supplementary material, which is available to authorized users.

## Introduction

Chemotherapy-induced diarrhoea (CID) is an important and commonly observed adverse event (AE) of particular relevance with 5-fluorouracil, irinotecan, capecitabine, anthracyclines, monoclonal antibody or small-molecule therapies [1–7]. The prevalence and severity may vary depending on chemotherapeutic regimen and dosage, but severe diarrhoea has been reported by up to 50% of treated patients [1]. It is usually managed symptomatically with antidiarrheal agents, diet modification and hydration [2]. However, in some patients it may influence their adherence to oral medication, lead to treatment delays or dose reduction, all of which can reduce the efficacy of treatment [8, 9]. CID also interferes with patients’ social and daily activities and quality of life (QoL), can increase psychological distress, and economic and carer’s burdens [10–13].

Accurate reporting is crucial for good management of CID. The widely adopted Common Terminology Criteria for Adverse Events (CTCAE) developed by the US National Cancer Institute (NCI), are the current standard for identification and grading of treatment-related AEs, including diarrhoea [14]. Although the CTCAE grading system is universal and important, it has limitations because it focuses primarily on the degree of medical intervention needed, and does not take into account patient’s perception of symptoms or impact on daily activities and QoL. Furthermore, the information is collected from medical records or by clinicians. Previous research has highlighted that there is low agreement between patients’ and clinicians’ AEs reporting, with clinicians frequently underestimating incidence and severity [15–17]. There is also poor concordance between patient-reported outcomes and clinical trial documentation, like case report forms or AE logs [18]. The increasing number of oral therapies now available for the treatment of cancer, raises another concern – that of adherence [19, 20]. Research suggests that many cancer patients struggle to take their medications as prescribed, with adherence often declining over time [20]. Medication factors (toxicity, AEs) were associated with nonadherence, therefore if CID is not managed well, patients may stop taking their medication, reducing potential treatment efficacy.

There is growing evidence that patient-reported outcome measures (PROs) provide important symptom data and are more sensitive in describing symptom burden compared to standard toxicity assessment tools. Many clinical trials incorporate PROs, usually generic instruments like the EuroQoL 5D-5L [21], or cancer-specific scales, including the Functional Assessment of Cancer Therapy-General (FACT-G) [22] or the European Organization for Research and Treatment of Cancer Quality of Life Questionnaire [23]. However, these commonly used PROs have little to no assessment of diarrhoea, and do not fully capture its impact on QoL. A symptom-specific subscale, the Functional Assessment of Chronic Illness Therapy for patients with Diarrhea (FACIT-D) was developed to be used alongside the FACT-G [24]. The FACIT-D contains 11 items to measure stool frequency, bowel control, incontinence, sleep disruption, emotional impact, and limitations of physical, social and sexual functioning. It has been used in international research, predominantly in trials examining the use of prophylactic drugs or other products in preventing or reducing CID [25–30]. Although the subscale measures QoL specific to diarrhoea, it lacks evaluation of symptom management, such as self-care and management strategies related to diet, nutritional supplements, and medication administration. The aim of this two-phase study was to design a measure that could be used alongside the FACIT-D, or separate from it, to monitor the presence and severity of CID and to capture some ways in which patients might control this when taking anticancer drugs. This paper describes the development and initial evaluation of this new tool, the Diarrhoea Management Diary (DMD).

## Methods

### Phase 1: development of the DMD

**Fig. 1:**
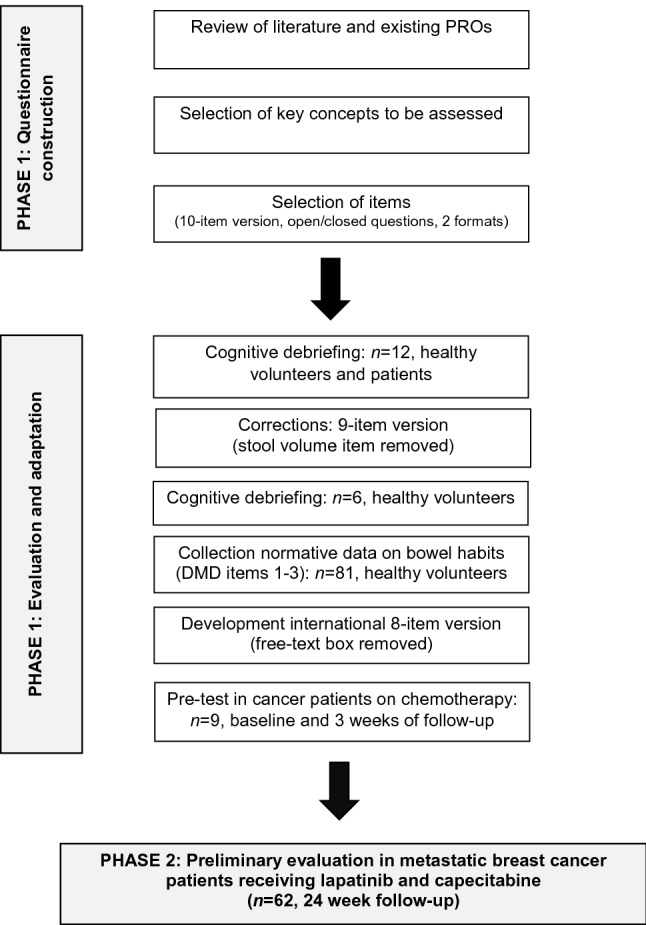
The process of the development of the Diarrhoea Management Diary (DMD)

Phase 1 followed the recommendations from ISPOR’s Good Research Practices Task Force Reports [31, 32]. An iterative process of instrument development was used that focused on questionnaire design and scaling, pilot testing and revision of preliminary versions (Fig. 1). To inform the selecting of items, a rapid concept-focused literature review [33] was conducted exploring information about CID and self-management strategies, and existing questionnaires of treatment/disease-related diarrhoea, or bowel dysfunction. Preliminary testing was carried out with an initial list of items to refine the draft instrument. Cognitive debriefing interviews were conducted in cancer patients with gastrointestinal symptoms, or bowel conditions (coeliac, inflammatory bowel disease) and controls who were selected by convenience sampling. An interview guide was used to assess instructions, interpretation of content, item relevance/order, word clarity, format and length.

### Phase 2: evaluation of the DMD

#### Study design and sample

The objective of phase 2 was to determine whether the DMD is easy to understand and appropriate for use in cancer patients. Data were collected in an international multicentre randomised trial investigating the prophylactic use of long-acting release octreotide on reducing diarrhoea associated with treatment for metastatic breast cancer (NCT02294786). Eligible patients were women aged 18 years or older with HER2-positive advanced or metastatic breast cancer which had progressed following prior therapy. Patients were randomised to receive octreotide (40 mg 7 days before chemotherapy and 28 days later) or no octreotide. All patients received combination therapy with lapatinib (1250 mg once daily continuously) and capecitabine (1000 mg/m^2^ twice daily on days 1–14 of a 21-day cycle) until disease progression.

#### Study procedures and measures

Self-reported diarrhoea, information on CID management, and treatment adherence was collected at baseline using the DMD (items 1–3 only) and on a weekly basis after the start of chemotherapy (Online Resource 1). QoL was evaluated at baseline and every 3 weeks (after each cycle) using the FACIT-D (version 4) [22, 24]. The FACIT-D comprises of the FACT-G with four subscales measuring physical, social, emotional and functional well-being, and the Diarrhoea Subscale (DS,11 items). There are five response options for each item with scores ranging from not at all to very much (scored 0–4). A total summary score is calculated by adding the subscale scores; total FACIT-D scores range from 0–152, and total DS scores range from 0–44. Higher values represent better QoL.

#### Statistical analyses

Only patient-reported data (DMD, FACIT-D) were analysed (a trial summary is available elsewhere) [34]. FACIT*-*D (total, DS subscale) scores were calculated for each time-point (baseline, 3, 6, 9, 12, 15, 18, 21 and 24 weeks) using the scoring algorithms provided, and missing data were treated according to scoring guidelines. Patterns of missing data were evaluated for both questionnaires. Demographic characteristics and PRO data were summarised using descriptive statistics: means and standard deviations (SD) for quantitative variables, and frequencies and percentages for categorical variables. Our CID definition included both increased frequency and altered consistency [35]. Self-reported diarrhoea on the DMD was defined as an increase in reported frequency of bowel movements and/or worsening in consistency (dichotomised as ‘hard/firm or quite soft’ to ‘very soft/loose or watery’) from baseline. The proportion of patients reporting diarrhoea (yes/no) was summarised for each time-point and the total number of diarrhoea episodes for each patient was calculated. A severity score was derived by dividing the total number of episodes by the number of time-points completed, and patients were divided into two categories: low/medium severity (i.e. reporting diarrhoea less than half of time on study) and high severity (i.e. reporting diarrhoea over half of time on study). Independent *t*-tests were performed to compare low/medium with high severity groups for differences in age and treatment duration.

To examine the sensitivity to change of the DMD capture of self-reported diarrhoea, we looked at the association between self-report of diarrhoea or not at each time-point (classified as described above) with meaningful change on the FACIT DS. We used a distribution-based approach to determine meaningful change in FACIT DS scores, with the threshold for change set at 0.5 SD change from baseline [36]. Patients were categorised as reporting meaningful decline, improvement or unchanged/stable at each time-point. Chi-square analyses (Fisher’s exact) were used to contrast the proportion of patients reporting meaningful decline or not in QoL (FACIT DS) with those self-reporting diarrhoea or not (DMD). All statistical analyses were conducted using IBM SPPS Statistics for Windows, Version 25.0 (IBM Corp., Armonk, N.Y., USA).

## Results

### Phase 1: development of the DMD

Literature searches were performed using various search terms (e.g. diarrhoea OR diarrhea, bowel movement, stools, cancer, chemotherapy, radiotherapy, assessment, questionnaire, PRO) and databases (e.g. Scopus, MEDLINE, Cochrane Library). A manual search within the selected papers and existing questionnaires was also conducted. The results were summarised and revealed a broad range of topics regarding CID and management strategies, and various instruments to assess diarrhoea in various medical conditions, like gastrointestinal diseases, bowel cancer and HIV. Figure 1 shows the development of a preliminary framework, which included the construction of a provisional list of questionnaire items. The draft DMD contained 10 items with open-ended and closed questions relating to stool frequency, consistency and volume (item 1–4); self-care and symptom management, including dietary changes (item 5–7); treatment adherence (item 8, 9); and, an open response option (item 10). Two DMD versions were developed, each with a different format. Both versions were used in cognitive debriefing with 7 patients and 5 controls (9/12 female; mean age 53.6 years, range 37–65), and were completed in counterbalanced order.

Analyses after iterative testing showed that the DMD item relevance, content interpretation, word clarity, and format or length were generally good. No additional items were suggested, but the item about stool volume was removed, and the response scales of two items extended. Other revisions consisted of layout changes. The revised DMD was administered to healthy volunteers (*n* = 6) for a small-scale field pre-test, and no further modifications were implemented.

Data were collected from a convenience sample of 81 healthy adults (50/81 female; mean age 46.3 years, range 20–66) to gather information about normative ranges for stool frequency and consistency (DMD items 1 to 3). The results were in line with published data [37–39] and showed that 95% had 1 to 3 bowel movements daily, mostly (93.7%) hard/firm or quite soft.

A DMD format with closed-ended questions was constructed for use in international research to enable easier quantification. The acceptability of this closed-format version was tested in a local teaching hospital as part of an audit. Nine cancer patients (6/9 female; 7/9 breast or bowel cancer; mean age 57.6 years, range 41–66) before the start of chemotherapy (items 1–3 only), followed by a weekly assessment for 3 consecutive weeks. They showed no problems of understanding the questions, and there were no missing values which might be a good indicator of acceptability.

The final version of the DMD has 8 items organised in three sections: (i) bowel habits (items 1–3); (ii) self-care and diarrhoea management (items 4–6, including 4 sub-items); and (iii) treatment adherence (items 7, 8). The DMD has no numerical scoring system, but changes over time for categorical and dichotomous responses can be charted either for each individual (showing diarrhoea onset and/or resolution) or for groups of patients, if used within a clinical trial setting. Cross-cultural translations were completed by the FACITtrans group for eight languages to use the measure in international research.

### Phase 2: evaluation of the DMD

#### Sample characteristics

Between December 2014 and April 2016, 62 patients from 17 centres in 5 countries were enrolled. Recruitment ended in September 2016, and the trial was stopped early due to futility (i.e. an interim analysis revealed that prophylactic octreotide did not result in a statistically significant or clinically meaningful reduction in occurrence of physician reported grade ≥ 2 diarrhoea) [34]. The median age was 57 years (range 33–81), and mean time since initial diagnosis to study entry was 4.3 years. All except one had cancer-related surgery, and all had prior systemic therapy. Thirty-seven (59.7%) patients completed 8 cycles of study treatment and 24 weeks follow-up. The most frequently recorded reason for study discontinuation was disease progression.

#### PRO completion

A total of 1220 DMDs (77.3% of the expected number of questionnaires) and 470 FACIT-Ds (80.1% of the expected number of questionnaires) were completed. Overall completion rates ranged from 98.4% at baseline to 59.7% at the end of study (Online Resource 2). In total, 60 patients (96.8%) completed both PROs at baseline and at least one subsequent time-point. Completion rates remained high until week 15 (71.0%). Over half of patients (*n* = 32; 51.6%) completed all PROs from baseline to week 24. Missing data were predominantly associated with study discontinuation (due to disease progression). Only one patient (1.6%) requested to stop the PRO completion due to a high burden.

Partial response (the occurrence of missing items) was found on 66 DMDs (from 28 patients) and 96 FACIT-Ds (from 34 patients), with respectively 85 (0.9%) and 282 (1.6%) item responses missing. Missing DMD data (main items only) were more frequent for questions that assessed diarrhoea management and self-care strategies (items 4–6; accounting for 55.3% of missing item responses). Missing item response on the FACIT-D was often observed on DS subscale items (146/282; 51.8%), or items related to sexual activity (81/282; 28.7%), and was treated according to the scoring guidelines which allows a subscale score to be prorated for missing items if greater than 50% of items are answered.

#### PRO analysis

A total of 58 patients were used for the PRO analysis; 3 patients were excluded because they had diarrhoea at baseline (≥ 6 stools daily) and 1 patient lacked post-baseline data. Before the first dose of octreotide and start of chemotherapy, 98.3% of patients had ≤ 2 daily stools and consistency was either hard/firm (47%) or quite soft (53%). The presence of self-reported diarrhoea after the start of chemotherapy was calculated for each patient separately. Table 1 shows bowel habits and occurrence of self-reported diarrhoea at follow-up (ranging from 42% to 67.9%). Eleven patients (19%) had persistent diarrhoea (diarrhoea at every time-points), and 6 (10.3%) reported no diarrhoea symptoms during treatment. Patients grouped by diarrhoea severity did not differ significantly in age or treatment duration (*p* > 0.05).

**Table 1:** Bowel movements and self-reported diarrhoea measured on the DMD during chemotherapy (*n* = 58)

Follow-up in weeks^a^	Wk1	Wk2	Wk3	Wk4	Wk5	Wk6	Wk7	Wk8	Wk9	Wk10	Wk11	Wk12
	*n* = 55	*n* = 56	*n* = 56	*n* = 56	*n* = 56	*n* = 54	*n* = 51	*n* = 50	*n* = 49	*n* = 44	*n* = 45	*n* = 44
Number stools per day^b^												
Median	2	2	1	2	2	2	2	2	2	1	2	2
Range	0–7	0–5	0- ≥ 8	0–7	0–7	0–7	0–7	0- ≥ 8	0- ≥ 8	0–5	0–7	0–7
Stool consistency^c^ (%)												
Hard/firm	9.1	3.6	14.3	5.4	5.4	7.3	9.8	8	6.1	9.1	8.9	9.1
Quite soft	67.3	60.7	51.8	62.5	62.5	65.5	66.7	56	67.3	77.3	68.9	59.1
Very soft/loose	12.7	30.7	26.8	30.4	30.4	21.8	19.6	30	20.4	13.6	20	25
Watery	10.9	5.4	7.1	1.8	1.8	5.5	3.9	6	6.1	0	2.2	6.8
Self-reported diarrhoea (%)	50.9	66.1	58.9	58.9	67.9	59.3	58.8	52	51	47.7	48.9	56.8
Diarrhoea every day^d^ (%)	48	50	37.5	33.3	31.6	19.4	50	38.5	48	38.1	36.4	48

Follow-up in weeks^a^	Wk13	Wk14	Wk15	Wk16	Wk17	Wk18	Wk19	Wk20	Wk21	Wk22	Wk23	Wk24
	*n* = 42	*n* = 42	*n* = 42	*n* = 39	*n* = 40	*n* = 40	*n* = 37	*n* = 38	*n* = 37	*n* = 37	*n* = 36	*n* = 35
Number stools per day^b^												
Median	1	1	2	1	1	1	1	1	1	1	2	2
Range	0–6	0–6	0–6	0–7	0–7	0–7	0–6	0–5	0–6	0–5	0–7	0–7
Stool consistency^c^ (%)												
Hard/firm	11.9	11.9	4.8	15.4	25	15	10.8	16.2	18.9	18.9	11.1	8.6
Quite soft	69	66.7	66.7	64.1	55	62.5	67.6	64.9	56.8	64.9	72.2	65.7
Very soft/loose	19	21.4	23.8	15.4	17.5	20	18.9	18.9	21.6	10.8	16.7	20
Watery	0	0	4.8	5.1	2.5	2.5	2.7	0	2.7	5.4	0	5.7
Self-reported diarrhoea (%)	47.6	50	54.8	51.3	47.5	47.5	48.6	42	45.9	45.9	58.3	57.1
Diarrhoea every day^d^ (%)	35	33.3	36.4	40	36.8	31.6	44.4	25	23.5	35.3	28.6	36.8

*Wk* week, *DMD* Diarrhoea Management Diary

^a^Numbers at follow-up do not equal 58 due to study attrition and non/incomplete response

^b^At baseline before starting treatment 98.3% had ≤ 2 daily stools (mean = 1.1, SD = 0.7)

^c^At baseline before starting treatment consistency was hard/firm in 47% and quite soft in 53%

^d^Percentage of patients who selected ‘every day’ on DMD item 2 (‘Over the past week how many days were typically like this?

Table 2 shows the proportions of patients with DMD grading of diarrhoea or not and meaningful improvement, deterioration or stable scores on the FACIT DS. The majority of patients showed a meaningful decline from baseline at each time-point. Because very few patients showed improvement at each time-point, the stable and improvement groups were collapsed for further exploratory analyses. Chi-square analyses (Fisher’s exact) showed significant associations between DMD grading of diarrhoea and meaningful decline on the DS at all follow-up time-points with the exception of week 9, suggesting that the DMD grading shows promising sensitivity to change in diarrhoea related QoL.

**Table 2:** Overview of self-reported diarrhoea, FACIT-D outcomes and meaningful change scores (*n* = 58)

Study time-points in weeks^a^	Baseline *n* = 58	Wk 3 *n* = 56	Wk 6 *n* = 54	Wk 9 *n* = 49	Wk 12 *n* = 44	Wk 15 *n* = 42	Wk 18 *n* = 40	Wk 21 *n* = 37	Wk 24 *n* = 35
No diarrhoea^b^, *n* (%)	58 (100)	23 (41.1)	22 (40.7)	24 (49.0)	19 (43.2)	19 (45.2)	21 (52.5)	20 (54.1)	15 (42.9)
Diarrhoea^b^, *n* (%)	–	33 (58.9)	32 (59.3)	25 (51.0)	25 (56.8)	23 (54.8)	19 (47.5)	17 (45.9)	20 (57.1)
FACIT-D, mean (SD)	115.3 (17.1)	113.7 (19.5)	108.6 (20.4)	111.2 (21.2)	112.2 (21.0)	109.9 (25.5)	113.0 (22.9)	114.8 (22.3)	115.8 (20.0)
range	77–147	69–151	64–151	57–147	74–148	45–148	58–148	64–148	70–148
DS score, mean (SD)	41.5 (3.6)	37.7 (6.8)	36.5 (6.6)	36.4 (7.6)	37.4 (5.6)	36.8 (7.3)	38.7 (5.8)	39.3 (5.4)	38.5 (5.6)
range	30–44	16–44	18–44	8–44	22–44	16–44	23–44	24–44	24–44
Improvement, *n* (%)	N/A	5 (8.9)	3 (5.4)	2 (4.3)	3 (7.0)	2 (4.8)	3 (7.9)	3 (7.9)	4 (11.8)
Stable, *n* (%)	N/A	18 (32.1)	14 (25.0)	11 (23.4)	15 (34.9)	11 (26.2)	15 (39.5)	14 (36.8)	9 (26.5)
Deterioration, *n* (%)	N/A	33 (58.9)	39 (69.6)	34 (72.3)	25 (58.1)	29 (69.0)	20 (52.6)	21 (55.3)	21 (61.8)
*X*^2^	N/A	4.50	5.01	0.49	7.29	7.63	18.03	6.36	5.38
*p*-value	–	0.034	0.025	.484	0.007	0.006	< 0.0001	0.012	0.020

*FACIT-D* the Functional Assessment of Chronic Illness Therapy for patients with Diarrhoea, *DS* FACIT-D Diarrhoea Subscale, *Wk* week, *DMD* Diarrhoea Management Diary

^a^Numbers at follow-up do not equal 58 due to study attrition and non/incomplete response

^b^Self-reported diarrhoea on the DMD was defined as an increase in reported frequency of bowel movements and/or worsening in consistency [35] (dichotomised as ‘hard/firm or quite soft’ to ‘very soft/loose or watery’) from baseline. At baseline before starting treatment 98.3% had ≤ 2 daily stools (mean = 1.1, SD = 0.7) and consistency was hard/firm in 47% and quite soft in 53%

#### Management and self-care strategies

Table 3 shows the use of management strategies for diarrhoea. In total, 24 patients (41%) tried one or more interventions, with over half (54%; 13/24) using just one strategy. Interventions were predominantly used within the first 9 weeks of treatment. Dietary change was the most frequently used approach (reported 83 times by 14 patients) and was successful (response options ‘a little’ and ‘quite a bit’) for most (84%) who tried this. Following a special diet (reported 52 times by 14 patients) was successful in 74%.

**Table 3:** Diarrhoea management and self-care strategies measured on the DMD (*n* = 58)

Follow-up in weeks^a^	Wk1 *n* = 55	Wk2 *n* = 56	Wk3 *n* = 56	Wk4 *n* = 56	Wk5 *n* = 56	Wk6 *n* = 54	Wk7 *n* = 51	Wk8 *n* = 50	Wk9 *n* = 49	Wk10 *n* = 44	Wk11 *n* = 45	Wk12 *n* = 44
Dietary changes^b^, *n* (%)	8 (14)	9 (15)	9 (16)	8 (14	6 (11)	7 (13)	5 (10)	3 (6)	5 (10)	1 (2)	2 (4)	2 (5)
Use of non-prescribed drugs^c^, *n* (%)	5 (9)	7 (12)	7 (12)	7 (13)	5 (9)	7 (13)	3 (6)	3 (6)	5 (10)	0	3 (7)	2 (5)
Contact/advice from HCPs^d^, *n* (%)	1 (2)	2 (4)	4 (7)	2 (4)	0	0	0	4 (8)	3 (6)	0	0	0
Reducing treatment^e^, *n* (%)	1 (2)	1 (2)	1 (2)	0	0	1 (2)	0	2 (4)	3 (6)	0	2 (4)	1 (2)
Discontinuing treatment^e^, *n* (%)	1 (2)	1 (2)	1 (2)	0	0	2 (4)	0	2 (4)	3 (6)	0	2 (4)	1 (2)

Follow-up in weeks	Wk13 *n* = 42	Wk14 *n* = 42	Wk15 *n* = 42	Wk16 *n* = 39	Wk17 *n* = 40	Wk18 *n* = 40	Wk19 *n* = 37	Wk20 *n* = 38	Wk21 *n* = 37	Wk22 *n* = 37	Wk23 *n* = 36	Wk24 n = 35
Dietary changes^b^, *n* (%)	1 (2.5)	1 (2.5)	2 (5)	0	2 (5)	2 (5)	1 (3)	1 (3)	1 (3)	2 (5)	2 (6)	3 (9)
Use of non-prescribed drugs^c^, *n* (%)	3 (7)	2 (5)	2 (5)	1 (2.5)	2 (5)	1 (2.5)	1 (3)	1 (3)	1 (3)	1 (3)	1 (3)	2 (6)
Contact/advice from HCPs^d^, *n* (%)	0	0	0	0	0	0	0	0	0	1 (3)	0	0
Reducing treatment^e^, *n* (%)	1 (2)	1 (2)	0	1 (3)	1 (3)	2 (5)	0	0	0	1 (3)	2 (6)	1 (3)
Discontinuing treatment^e^, *n* (%)	0	0	0	0	0	2 (5)	0	0	0	0	0	0

^a^Numbers at follow-up do not equal 58 due to study attrition and non/incomplete response

^b^Avoiding certain foods and/or following a special diet

^c^Use of medication not prescribed by hospital doctor (i.e. drugs to reduce bowel frequency or cramping/pain)

^d^Other than hospital doctor (e.g. general practitioner, pharmacist)

^e^Oral chemotherapy (i.e. lapatinib, capecitabine)

One in four patients (15/58) used non-prescribed medication for bowel control. All had used drugs to reduce frequency of bowel movements, which was successful in 73%. Using medication to relieve cramping or pain had a higher success rate (78%), but was reported less frequently. Medication to reduce bowel frequency or cramping and pain was ineffective in respectively 10% and 21% of cases.

Seven patients (12%) contacted a HCP other than their hospital doctor for advice. This was often a general practitioner (8/16) with most receiving advice on diet, fluids intake and rest. No advice was provided about skin care or use of supplements.

One in eight patients (12%) adjusted or stopped oral chemotherapy to control diarrhoea symptoms, most frequently during the first 12 treatment weeks. Dose reductions (on most days of the week) were reported 22 times in just under half (45.5%) of patients. Seven patients reported that they completely stopped treatment; 40% on most days of the week.

## Discussion

Diarrhoea is a common debilitating AE of anticancer treatment, often affecting QoL and sometimes long-term outcomes [40, 41]. In cancer clinical trials AEs reporting is mandatory to understand treatment toxicities and monitor patient safety, but this is usually done by investigators rather than patients [42]. This paper described the development and initial evaluation of the DMD, a scale that measures symptoms and self-care and management strategies of diarrhoea, including adherence to treatment. The content was developed by literature review and direct patient feedback. During the cognitive debriefing process, the DMD was easily understood by controls and cancer patients, and relevant to their experiences and management of diarrhoea. The final 8-item scale was administered to 62 patients receiving lapatinib and capecitabine for metastatic breast cancer in an international multicentre randomised trial. A change in the frequency and stool consistency was observed in the majority of patients, with almost one in five patients having CID at all follow-up time-points. DMD diarrhoea grading was associated with clinically meaningful change on the diarrhoea subscale of the FACIT-D, indicating that our scale was robust enough to monitor change in individual patients over time.

There is increasing recognition that integration of PROs into both research and clinical care is essential for the delivery of patient-centred care. PROs can provide meaningful data about symptoms, treatment tolerance and QoL, and are important to clinicians and patients making informed treatment choices. To improve the accuracy of clinician-reported AEs and address the growing need for PROs in cancer trials, the NCI developed a PRO version of the CTCAE, the PRO-CTCAE [43]. For each AE assessed, PRO items assess one of the following attributes: frequency, severity, interference with usual or daily activities, presence/absence, and amount. The PRO-CTCAE has good validity and reliability and now serves as a companion to the CTCAE using a software platform [44, 45]. Although this approach may improve AE reporting in large clinical trials, diarrhoea on PRO-CTCAE is only assessed by the frequency of loose or watery stools (as opposed to an increase in stools on the CTCAE). Severity of symptoms (important to monitor changes over time) and interference with daily living are not evaluated, nor are impact on QoL, self-care, symptom management or treatment adherence. Inclusion of additional PROs to cover these areas is therefore recommended.

It is also well known that early recognition and management of diarrhoea is essential to prevent dose reduction or treatment discontinuation. However, management of CID varies greatly amongst medical providers, and research on self-care methods and symptom management, like dietary interventions or nutritional education has been limited [46–49]. A review of assessment and management of chemotherapy-related toxicities in patient with common types of cancer showed a lack of research papers addressing symptom management interventions or self-care strategies for diarrhoea [46]. Our study outcomes showed that self-care methods were primarily reported by patients during the first weeks of treatment, and dietary change was the most common strategy to control diarrhoea. Almost a quarter of patients used an exclusion diet (e.g. avoiding or limiting spicy or fatty food, dairy products) or followed a special diet, such as the BRAT diet (i.e. banana, rice, apples, toast diet), which was successful in the majority of patients who tried this. Having the additional information of possible favourable effects of certain self-care methods could be useful and could inform patient education about CID.

## Strengths and limitations

Our study has several strengths: this newly developed instrument includes assessment of frequency and consistency of bowel movements and habits, as well as a thorough evaluation of the use of self-care and management strategies for diarrhoea symptoms, and permits some measure of adherence to treatment. We used a sequential design for development, evaluation and testing patients receiving treatment for metastatic breast cancer. Preliminary data from this multicentre international trial showed that the DMD has the ability to monitor symptoms frequently (weekly) over a longer period of time, with relatively low nonresponse rates. The scale is available in English and was translated into eight other languages, permitting use in international oncology clinical trials.

The main limitation of this study is that validity data of the DMD is restricted to content validity as the final sample size and response to individual items was small. Also, evaluation of the practicability was conducted in a relatively small sample of metastatic breast cancer patients only, and the results should be interpreted with this in mind. Future research needs to demonstrate further validity and reliability, and ascertain whether the scale can be used in more diverse cancer populations, including patients receiving non-oral anticancer therapies, such as parenteral chemotherapy, some types of immunotherapy and radiotherapy.

We should also mention here that after initiation of our study another PRO for CID was developed: the Systemic Therapy Induced Diarrhoea Assessment Tool [50]. This scale assesses onset and duration of diarrhoea, including diarrhoea-associated symptoms (e.g. abdominal discomfort, urgency), and evaluates impact on QoL (activities of daily living, energy levels, mood, social/family life). However, measurement of management strategies is limited to use of antidiarrhoeal medication, and items addressing treatment adherence are lacking.

## Conclusion

The DMD is a brief measure developed by means of qualitative and quantitative research, including patient feedback on several versions of the tool. The DMD was designed to measure diarrhoea, and self-care and management strategies in adults receiving anticancer treatment. Although further psychometric testing in other populations is recommended, outcomes reported here provide preliminary evidence of promising discriminative ability.

## Electronic supplementary material

Below is the link to the electronic supplementary material.

Supplementary file1 (DOCX 35 kb)

Supplementary file2 (PDF 163 kb)

## Data Availability

The datasets during and/or analysed during the current study are available from the corresponding author on reasonable request.
