# Supplementary material for: The development and initial evaluation of the Diarrhoea Management Diary (DMD) in patients with metastatic breast cancer
Source: Breast Cancer Res Treat. 2020 Jul 27;183(3):629–38. doi: 10.1007/s10549-020-05798-w (PMC7497672; doi:10.1007/s10549-020-05798-w)
Supplement: Supplementary file 1 — Supplementary file1 (DOCX 35 kb) [file 10549_2020_5798_MOESM1_ESM.docx]

**Breast Cancer Research and Treatment**

**The development and initial evaluation of the Diarrhoea Management Diary (DMD) in patients with metastatic breast cancer**

Helena Harder^1^, Valerie M. Shilling^1^, Shirley F. May^1^, David Cella^2^, Peter Schmid^3^ and Lesley J. Fallowfield^1^

^1^ Sussex Health Outcomes Research and Education in Cancer (SHORE-C)

Brighton and Sussex Medical School

University of Sussex

Brighton, UK

^2^ Department of Medical Social Sciences

Feinberg School of Medicine

Northwestern University

Chicago, IL, USA

^3^ Centre for Experimental Cancer Medicine

Barts Cancer Institute

Queen Mary University London

London, UK

Corresponding author:

Dr Helena Harder

T +44 (0)1273 873 019

F +44 (0)1273 873 022

[h.harder@sussex.ac.uk](mailto:h.harder@sussex.ac.uk)

**Diarrhoea Management Diary (DMD)**

*version 9, © 2014 SHORE-C, University of Sussex*

*This questionnaire will help the study team to understand more about how you manage treatment related diarrhoea. Your responses are strictly confidential. Please answer every question as it applies to you in the* ***past 7 days*** *by marking the appropriate box* 🗷

**1.** Over the past week how many bowel movements (include diarrhoea or loose watery stools) did you have on a **typical** day:

none□ 1□ 2□ 3□ 4□ 5□ 6□ 7□ 8 or more□

**2.** Over the past week how many days were typically like this?

every day□ almost every day (5-6 days)□ some days (3-4 days)□

**3.** Over the past week were your bowel movements usually:-

hard/firm□ quite soft□ very soft/loose□ watery□

**4.** Over the past week have you changed your diet to try and help with the diarrhoea?

no□ (go to question 5) yes□ *If yes, go to question 4a*

**4a.** What were these and how successful were they? (*please mark all that apply*)

*i)* avoid certain foods □

How successful was this?

not at all □ a little □ quite a bit □ very successful □

*ii)* follow a special diet □

How successful was this?

not at all □ a little □ quite a bit □ very successful □

**5.** Over the past week did you take any extra medicines **not prescribed** by the hospital doctors to try and help with the diarrhoea?

no□ (go to question 6) yes□ *If yes, go to question 5a*

**5a.** What were these and how successful were they? (*please mark all that apply)*

*i)* medicines to reduce frequency of bowel movements □

How successful was this?

not at all □ a little □ quite a bit □ very successful □

*ii)* medicines to relieve the cramping/pain □

How successful was this?

not at all □ a little □ quite a bit □ very successful □

**6.** Over the past week have you contacted any health care professional other than the hospital doctors/nurses to discuss the diarrhoea?

no□ (go to question 7) yes□ *If yes, go to question 6a/6b*

**6a)** Who was this? *(please mark all that apply)*

GP □ practice nurse □ pharmacist □ other □

**6b)** What did they advise and did you try this? *(please mark all that apply)*

*Did you try this? 🡪* no yes

*i)* increase fluid intake □ □ □

*ii)* change of diet □ □ □

*iii)* use of dietary supplements □ □ □

*iv)* use of herbal remedies □ □ □

*v)* use of anti-diarrhoeal medicines □ □ □

*vi)* help with anal soreness/skin damage □ □ □

*vii)* rest and reduce normal activity □ □ □

*viii)* other □ □ □

**7.** Over the past week have you reduced the number of your anti-cancer tablets to try and help with the diarrhoea?*

never□ once or twice□ 3 to 4 days□ most days this week□

**8.** Over the past week have you ever completely stopped taking your anti-cancer tablets to try and help with the diarrhoea?*

never□ once or twice□ 3 to 4 days□ most days this week□

** Please only answer this question if you are receiving oral therapy (i.e. tablets or capsules) to treat your cancer*

**Thank you for completing this questionnaire**
