# Supplementary material for: The development and initial evaluation of the Diarrhoea Management Diary (DMD) in patients with metastatic breast cancer
Source: Breast Cancer Res Treat. 2020 Jul 27;183(3):629–38. doi: 10.1007/s10549-020-05798-w (PMC7497672; doi:10.1007/s10549-020-05798-w)
Supplement: Supplementary file 2 — Supplementary file2 (PDF 163 kb) [file 10549_2020_5798_MOESM2_ESM.pdf]

Breast Cancer Research and Treatment

The development and initial evaluation of the Diarrhoea Management Diary (DMD) in patients with metastatic breast cancer

Helena Harder[1], Valerie M. Shilling[1], Shirley F. May[1], David Cella[2], Peter Schmid[3] and Lesley J. Fallowfield[1]

[1] Sussex Health Outcomes Research and Education in Cancer (SHORE-C)
Brighton and Sussex Medical School
University of Sussex
Brighton, UK

[2] Department of Medical Social Sciences
Feinberg School of Medicine
Northwestern University
Chicago, IL, USA

[3] Centre for Experimental Cancer Medicine
Barts Cancer Institute
Queen Mary University London
London, UK

Corresponding author:
Dr Helena Harder
T +44 (0)1273 873 019
F +44 (0)1273 873 022
h.harder@sussex.ac.uk

Supplementary Table. PROs completion rates from baseline to end of study (n=62)

Study time-points	DMD<br>n (%)	FACIT-D<br>n (%)
Baseline	61 (98.4)	62 (100)
Week 3	60 (96.8)	60 (96.8)
Week 6	58 (93.5)	59 (95.2)
Week 9	51 (82.3)	53 (85.5)
Week 12	46 (74.2)	47 (75.8)
Week 15	44 (71.0)	44 (71.0)
Week 18	41 (66.1)	41 (66.1)
Week 21	39 (62.9)	40 (64.5)
Week 24	37 (59.7)	37 (59.7)
